# Understanding the Natural Progression of Spina Bifida: Prospective Study

**DOI:** 10.2196/resprot.7739

**Published:** 2017-09-14

**Authors:** Judy Thibadeau, Matthew R Reeder, Jennifer Andrews, Katherine Ong, Marcia L Feldkamp, Sydney Rice, Ann Alriksson-Schmidt

**Affiliations:** ^1^ National Center on Birth Defects and Developmental Disabilities Centers for Disease Control and Prevention Atlanta, GA United States; ^2^ Department of Pediatrics Division of Medical Genetics University of Utah Salt Lake City, UT United States; ^3^ Department of Pediatrics University of Arizona Tucson, AZ United States; ^4^ Skane University Hospital Lund Sweden

**Keywords:** spina bifida, natural history, birth defect, disability, surveillance, recruitment

## Abstract

**Background:**

Spina bifida (SB) is monitored through birth defects surveillance across the United States and in most developed countries. Although much is known about the management of SB and its many comorbid conditions in affected individuals, there are few systematic, longitudinal studies on population-based cohorts of children or adults. The natural history of SB across the life course of persons with this condition is not well documented. Earlier identification of comorbidities and secondary conditions could allow for earlier intervention that might enhance the developmental trajectory for children with SB.

**Objective:**

The purpose of this project was to assess the development, health, and condition progression by prospectively studying children who were born with SB in Arizona and Utah. In addition, the methodology used to collect the data would be evaluated and revised as appropriate.

**Methods:**

Parents of children with SB aged 3-6 years were eligible to participate in the study, in English or Spanish. The actual recruitment process was closely documented. Data on medical history were collected from medical records; family functioning, child behaviors, self-care, mobility and functioning, and health and well-being from parent reports; and neuropsychological data from testing of the child.

**Results:**

In total, 152 individuals with SB were identified as eligible and their parents were contacted by site personnel for enrollment in the study. Of those, 45 (29.6%) declined to participate and 6 (3.9%) consented but did not follow through. Among 101 parents willing to participate, 81 (80.2%) completed the full protocol and 20 (19.8%) completed the partial protocol. Utah enrolled 72.3% (73/101) of participants, predominately non-Hispanic (60/73, 82%) and male (47/73, 64%). Arizona enrolled 56% (28/50) of participants they had permission to contact, predominately Hispanic (18/28, 64%) and male (16/28, 57%).

**Conclusions:**

We observed variance by site for recruitment, due to differences in identification and ascertainment of eligible cases and the required institutional review board processes. Restriction in recruitment and the proportion of minorities likely impacted participation rates in Arizona more than Utah.

## Introduction

Spina bifida (SB) is a neural tube defect that occurs in the first month after conception and involves a defect of variable severity in the developing spinal cord [1-3]. The term SB is an umbrella term and includes different types of spinal cord defects, of which myelomeningocele is the most frequent and the most involved. SB is monitored through birth defects surveillance across the United States and in most developed countries. A concerted public health emphasis on primary and secondary prevention has been associated with a decreased birth prevalence and improved health outcomes in individuals with SB [4]. In the United States, the estimated prevalence is 2-3 per 10,000 live births [5,6]. Although much is known about the management of SB and its many comorbid conditions in affected individuals, there are few systematic, longitudinal studies on population-based cohorts of children or adults. The natural history of SB across the life course of persons with this condition is not well documented [7,8].

Health, developmental, and school problems occur on a continuum for children with SB. Comorbidities (eg, hydrocephalus) are frequent [9] and because many body systems (eg, muscular/skeletal, renal/urinary) are affected simultaneously, SB is a complex condition to manage and treat. This complexity can be further compounded by the development of secondary conditions such as frequent urinary tract infections, pain, and depression [10,11]. Although more significant cognitive deficits may be apparent before the age of 3 years, the subtle learning and language problems common in children with SB [12] may not be identified by public school systems until children are 8-10 years old [13]. Assessment of learning and language problems may not occur until children are significantly behind their peers academically. Given that children with SB have such a high rate of school problems [14,15], the possibility of earlier identification (ie, ages 3-6 years) may allow for earlier academic and language interventions that enhance the academic performance and improve the developmental trajectory of the condition for these children.

In adulthood, individuals with SB have markedly wide ranges of outcomes in terms of physical function, social participation, and quality of life. Outcomes vary from full employment, successful relationships, and independent living (the typical goals of adulthood) to social isolation, depression, and under- or unemployment [16-18]. These outcomes, while partially determined by underlying health-related and cognitive issues (eg, shunt revisions, incontinence, mobility, challenges with executive function), are greatly influenced by the lived experience of the individual and by the environmental responses to the condition.

In 2010, the Centers for Disease Control and Prevention (CDC) announced a funding opportunity for a cooperative agreement entitled *A Prospective Assessment of the Development, Health, and Condition Progression in Young Children with Spina Bifida*. The purpose of this announcement was to assess the development, health, and condition progression by prospectively studying children who were born with SB. In addition, the methodology used to collect the data would be evaluated and revised as appropriate. The approach and methodology to be used in this project was informed by a pilot project that was conducted in Georgia [7]. The funded applicants, Arizona (university) and Utah (health department) worked in collaboration with CDC to refine and finalize the research protocol. In this first publication since the project’s conclusion, we describe and evaluate the methodology and recruitment process of the study.

## Methods

### Study Protocol

Prior to this study, CDC researchers and collaborators completed a pilot project to inform the current larger study [7]. The protocol used in the CDC pilot study served as a framework for the funding announcement. Because Utah and Arizona submitted separate applications in response to the funding announcement, a unified protocol was developed by both sites in the first year of the study. The two sites were tasked with identification, location, and recruitment of English- or Spanish-speaking parents with children with SB (International Classification of Diseases, Ninth Revision, Clinical Modification, codes 741.0, spina bifida with hydrocephalus and 741.9, spina bifida without hydrocephalus) born between September 1, 2004, and August 31, 2009, residing in one of the two catchment areas. Individuals living in nearby states were also recruited, but only if they attended an SB clinic at the participating sites. The birth date range was selected to ensure children were between 3 and 6 years at the time of enrollment in the study, which allowed the collection of extensive baseline data on health status, social, and cognitive development, of the children prior to entering school. Each child’s parent was required to be over 18 years old and able and willing to sign the consent forms. Institutional review board (IRB) approvals were obtained separately at the two sites. Recruitment began in May 2011 and ended in September 2013.

### Identification of Eligible Participants and the Recruitment Process

Children with birth defects were first identified from surveillance systems from both sites. IRBs at each site differed in how they allowed potential participants to be identified, contacted, and recruited; therefore, the recruitment processes varied between the two sites. In Utah, children with SB were identified using population-based statewide surveillance data from the Utah Birth Defect Network. Surveillance data included demographic and diagnostic information to determine case eligibility. All parents of eligible children were sent a recruitment letter from the Utah Department of Health introducing them to the study and inviting them to participate. Follow-up phone calls were made by the study coordinator approximately 7 days after the recruitment letter was sent to assess the parents’ interest in participating in the study. Parents of eligible children with SB who were attending the SB clinic during the study period were also invited by the SB clinic staff to participate. Occasionally, eligible children with SB and their parents traveling from Utah’s surrounding states (eg, Idaho, Wyoming, and Nevada) to attend the SB clinic in Salt Lake City were invited to participate. Staff in Utah were not restricted in contact attempts or recruitment by their local IRB.

In Arizona, eligible children with SB were identified through the birth defects monitoring program, hospital discharge databases, SB multispecialty clinics, and the primary children’s hospitals in Tucson and Phoenix to assemble the eligible population, including health and demographic characteristics of the children. The IRB for this site did not permit research staff to contact any patients without expressed consent; therefore, recruitment letters, emails, and Web announcements were sent advertising the study and providing contact information for self-enrollment through the Arizona Spina Bifida Association and from the Children’s Rehabilitation Services program (Medicaid-funded program for children with physical disabilities). Staff at the Phoenix and Tucson multispecialty SB clinics were allowed to make phone calls to parents of active patients informing them about the study and requesting permission for research staff to contact them. Active recruitment was also performed during scheduled visits to clinics at the Tucson location. However, the IRB at the Phoenix location with the largest SB population only permitted making flyers available to potential participants and did not permit any direct recruitment. The primary IRB approval limited the contact with any family to three phone calls at each stage in the recruitment process.

### Procedures and Measures Included

Parents were given two options to participate: (1) an in-person clinic visit with the child and family to complete neuropsychological assessments and parent surveys (ie, Full Protocol), or (2) a phone survey with mailed questionnaires to the parents (ie, Partial Protocol). Parents also had the option of a mailed questionnaire that could be completed and returned instead of either the in-person assessment or the phone interview. In all options, parents were asked consent to release their child’s medical records for medical record abstraction. Medical record abstraction was performed at both sites to obtain detailed data on clinic visits and hospitalizations, surgeries, growth, and comorbid conditions. Medical record data at each site were stripped of personal identifiers and transmitted to the CDC for pooling into a central dataset.

The in-person visits to the clinics were conducted in either English or Spanish and took 2-3 hours to complete; the phone survey lasted approximately 30 minutes. Because of the length of time associated with the in-person clinic visit, study appointments had to be scheduled on a day other than the child’s regular SB clinic visit. Gift cards of US $50 were given to parents who participated in the full protocol as compensation for travel and time, and US $25 gift cards were given to those who participated in the phone survey.

The survey included 120 items and covered topics regarding the child’s medical issues, development and learning, nutrition and physical growth, mobility and functioning, general health, and family demographic information. Although most of the survey items were created in the pilot project completed prior to the current study (with modifications for this specific study), many of the more generic items in the survey have previously been used in large national surveys, such as the Youth Risk Behavior Survey and the National Early Intervention Longitudinal Study. Parents who chose a phone interview were mailed the consent forms and medical records release form as well as five of the six self-administered questionnaires and were asked to complete them at home and send them back in the postage-paid envelope provided. One of the parent surveys (the Pediatric Evaluation Disability Index [PEDI]) was not mailed to the participants because the investigators considered it too difficult for parents to complete on their own. After the consent forms and surveys were completed and returned to the study coordinator, parents were contacted to schedule a time to participate in the phone survey.

At the beginning of each in-person study visit, the consent and parental permission forms and a medical record release form were presented to the parent and any questions or concerns were addressed. After the parent reviewed and signed these documents, the pediatric psychologist escorted the child into a separate room for neuropsychological testing. Parents were typically not in the exam room unless parental attendance was warranted according to the professional judgement of the clinician. The psychologist administered five assessments: the *Bracken Basic Concept Scale*‒Receptive (BBCS-R) [19], the *Differential Abilities Scale* 2^nd^ Edition (DAS-2) [20], the NEuroPSYchological Assessment (NEPSY) II [21], the *Peabody Picture Vocabulary Test*, 4^th^ Edition [22], and the *Wide Range Assessment of Visual Motor Abilities* (WRAVMA) [23]. For Spanish-speaking participants, all documents, including the consent, parental permission, and medical records release forms, parent interview, parent surveys, and three of the five child assessments (DAS-2, BBCS-R, and WRAVMA) were conducted in Spanish. In Utah, a translator was available for appointments with Spanish speakers to assist in the completion of the study documents, the parent interview, and the parent surveys and administering the battery of neuropsychological assessments used in the study. In Arizona, which has a large Hispanic population, all appointments were conducted by bilingual professionals. While the child was completing testing, the study coordinator administered the study survey to the parents, which was the same questionnaire used in the phone survey. Parents were asked to complete six self-administered questionnaires addressing family functioning, child behaviors and personality, self-care, mobility, and functioning, and health and well-being: the *Adaptive Behavior Assessment System*, 2^nd^ Edition [24]; the *Behavior Assessment System for Children*, 2^nd^ Edition [25]; the *Behavior Rating Inventory* [26]; the *Child Health Questionnaire* [27]; McMaster Family Assessment [28]; and *PEDI* [29]. The battery of assessments and questionnaires is provided in Table 1.

**Table 1:** Key characteristics of the instruments used in the prospective study of spina bifida in children, 2011-2013.

Instrument		Age range	Items/ subtests	Domains tested	Administration	Language
**Parent reported**						
	Adaptive Behavior Assessment System 2^nd^ Edition	0-89 yrs	241 items	Daily living skills 10 skill areas; Domains (1) social, (2) practical, & (3) conceptual	In-person, Mail	English, Spanish
	Behavior Assessment System for Children 2^nd^ Edition Parent Rating Scales-Preschool	2-5 yrs	134 items	Behavior and self-perceptions of children and young adults ages 2-25 years. Only the Parent Rating Scales were included in this project, which measures adaptive and problem behaviors in the community and home setting.	In-person, Mail	English, Spanish
	Behavior Assessment System for Children 2^nd^ Edition Parent Rating Scales-Child	6-11 yrs	160 items			
	Behavior Rating Inventory of Executive Function - Preschool Version	2-5 yrs	63 items	Executive Function Subscales: (1) emotional control, (2) shift, (3) inhibit, (4) working memory, & (5) plan/organize Indices: (1) inhibitory self-control, (2) flexibility, & (3) emergent metacognition	In-person, Mail	English, Spanish
	Behavior Rating Inventory of Executive Function	5-18 yrs	86 items			
	Child Health Questionnaire	5-18 yrs	50 items	Quality of life instrument measuring 14 unique physical and psychosocial concepts. The parent form (50 items) was used for this project	In-person, Mail	English, Spanish
	McMaster Family Assessment Device (FAD)		12 items	The 12-item general functioning scale of the FAD was used for this study. Both unhealthy family functioning (negative) and healthy family functioning (positive) items are included	In-person, Mail	English, Spanish
	Pediatric Evaluation of Disability Inventory	0.5-7.5 yrs	217 items	Functional abilities. Subdomains: (1) self-care, (2) mobility, & (3) social function. Parts: (1) functional skills, (2) caregiver assistance, & (3) modifications	In-person	English, Spanish
	Study Survey		120 items	Project-specific questionnaire containing items in six domains: (1) medical concerns, (2) development & learning, (3) nutrition & physical growth, (4) mobility & functioning, (5) general health, & (6) family demographics	In-person, Telephone, Mail	English, Spanish
**Psychologist administered**						
	BBCS-3:R	2:6-7:11 yrs	5 subtests	The School Readiness Composite was the only assessment from the BBCS-3:R used to assess children’s knowledge of those readiness concepts that parents and preschool and kindergarten teachers traditionally teach children in preparation for formal education. The subtests included the following: colors, letters, numbers/counting, sizes/comparisons, and shapes.	In-person	English, Spanish
	DAS-2	2:5-17:11 yrs	7 subtests	Cognitive abilities, 7 core subtests from early years battery: (1) verbal comprehension, (2) picture similarities, (3) naming vocabulary, (4) recall of objects, (5) pattern construction, (6) matrices, & (7) copying	In-person	English, Spanish
	NEPSY II	3-16:11 yrs	3 subtests	The Comprehension of Instructions and the Word Generation subtests from the Language domain and the Sentence Repetition from the Memory and Learning domain were the only subtests administered	In-person	English
	Peabody Picture Vocabulary Test 4^th^ Edition	2-6+	228 items	Measure of receptive vocabulary; included 228 test items each consisting of 4 full-color pictures as response options on a page	In-person	English
	WRAVMA	3-17 yrs	2 subtests	Visual-motor integration; WRAVMA matching visual-spatial subtest; WRAVMA pegboard fine-motor subtest	In-person	English, Spanish

The Arizona IRB did not require consent for medical record abstraction of eligible cases, whereas the Utah IRB did. The medical records of consented children in Utah, and eligible children in Arizona with or without consent, were abstracted at each site. Prior to the start of the project, the two sites and CDC agreed on a number of data elements to collect from the medical records. This included birth and mortality data (if applicable), as well as demographic information (ie, insurance status at birth, maternal and paternal ages at birth, race/ethnicity, education, gravidity, plurality, marital status, and occupation status), newborn hearing evaluation results, SB level of lesion and type, visual acuity measurements, conditions secondary to SB, and growth parameters. Additionally, information was collected regarding clinic visits (ie, clinic type, provider, and visit reason), hospitalizations (ie, admit/discharge dates, hospitalization reason, and discharge diagnosis codes/text), and surgical history (ie, dates, surgery type, reason, and procedural and diagnosis codes). Medical record data from both sites were entered into an Access database created by the Arizona team specifically for the project. An abstraction manual was created to ensure uniformity and consistency among abstractors at both sites.

## Results

The distributions of gender, race/ethnicity, type of primary insurance, and year of birth among children who participated and those who did not at each site are presented in Table 2. In Utah, there were no differences between participants and nonparticipants on these variables. In Arizona, participants were more likely to be Hispanic (*P*=.011, Fisher’s Exact Test). They were also more likely to have Medicaid as their primary insurance (*P*<.013, Fisher’s Exact Test).

In Utah, recruitment letters were sent to all 92 parents of eligible children identified in the birth defects surveillance system. An additional 27 recruitment letters were re-sent either because the initial letter came back as undeliverable or the parent stated that they did not receive it when the study coordinator called to follow up. Of the 92 parents, 52 participated in the full protocol, 13 in the partial protocol, and 27 declined. Of the 10 eligible children who were identified from the SB clinic but not born in Utah and who were invited by the SB clinic director, 7 participated in the full protocol, 1 in the partial protocol, and 2 did not respond. Medical records of 70 of the 73 who participated in either the partial or full protocol were abstracted. For Utah, participants who completed the full protocol did not differ from those who completed only the partial protocol on gender, race/ethnicity, insurance type, and year of birth.

**Table 2:** Descriptive information for participants and nonparticipants, by site, in the prospective study of spina bifida in children, 2011-2013^a^.

		Arizona, n (%)			Utah, n (%)		
		Partial (n=6)	Complete (n=22)	No participation (n=140)	Partial (n=14)	Complete (n=59)	No participation (n=29)
**Gender**							
	Male	11 (50)	5 (83)	71 (51)	8 (11)	39 (53)	16 (55)
	Female	11 (50)	1 (17)	68 (49)	6 (8)	20 (27)	11 (38)
	Ambiguous			1 (1)	—		—
	Missing	—	—		—		2 (7)
**Race/Ethnicity**							
	White	7 (32)	1 (17)	59 (42)	14 (19)	46 (63)	17 (59)
	Hispanic	13 (59)	5 (83)	57 (41)	—	10 (14)	9 (31)
	Other	2 (10)	—	24 (17)	—	3 (4)	1 (3)
	Missing	—	—		—	—	2 (7)
**Primary insurance**							
	Private insurance	2 (9)	0 (0)	14 (10)	2 (3)	12 (16)	—
	Medicaid/ Federal	11 (50)	3 (50)	32 (23)	2 (3)	12 (16)	—
	Other	1 (5)	—	94 (67)	—	—	
	Missing	8 (37)	3 (50)		10 (14)	35 (48)	29 (100)
**Year of birth**							
	2004	—	—	8 (6)	—	1 (1)	2 (7)
	2005	3 (11)	—	33 (24)	5 (7)	12 (16)	5 (17)
	2006	1 (4)	4 (14)	27 (19)	2 (3)	10 (14)	6 (21)
	2007	1 (4)	7 (25)	30 (21)	1 (1)	19 (26)	4 (14)
	2008	1 (4)	7 (25)	24 (17)	4 (5)	10 (14)	6 (21)
	2009	—	4 (4)	18 (13)	2 (3)	7 (10)	6 (21)

^a^Percentages may not add to 100% due to rounding.

**Figure 1:**
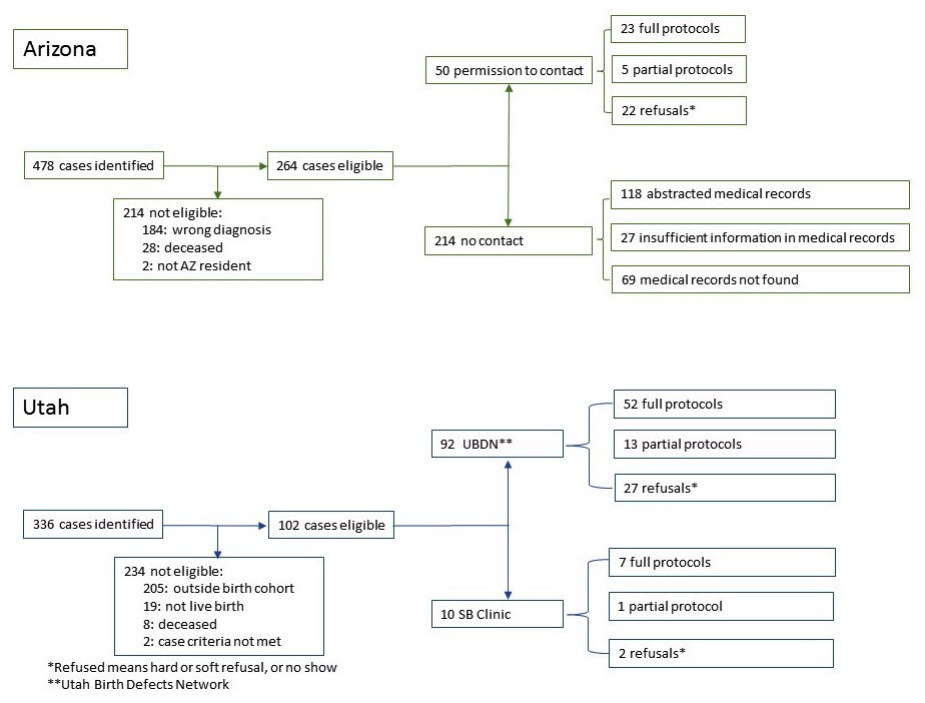
Recruitment flow chart.

Of the 264 eligible children identified in Arizona, permission was granted by 50 families for the research staff to contact them directly. Of these, 23 participated in the full protocol and 5 in the partial protocol. Six consented to participate but did not participate in the study, and 16 declined. Because letters were sent through partners and not directly by the research team, the Arizona site staff could not collect information on the nonenrollees. It is assumed that the remaining 214 cases did not want to respond or the research staff had the wrong addresses. Medical records were abstracted for surveillance for the 214 cases, 96 of which had little or no medical record information and were assumed to live outside the catchment area. A flow chart of the results of the recruitment process is presented in Figure 1. For Arizona, there were no differences between full and partial protocol participants on gender, insurance type, and race/ethnicity. However, the two groups differed on year of birth in that all of the participants born in 2005 completed partial protocol and all of the participants born in 2009 completed full protocol (*P*=.022, Fisher’s Exact Test).

In total, 152 parents of eligible children were directly contacted by the research staff for enrollment in the study. Of those, 45 (29.6%) declined to participate, and 6 (3.9) consented but did not follow through with the study. Of the 101 parents who agreed to participate, 82 (81.1%) participated in the full protocol and 19 (18.8%) participated in the partial protocol.

## Discussion

### Principal Findings

The aim of this project was to explore methods piloted by CDC to collect health, cognitive, and social development information for children with spina bifida by identifying eligible children in Arizona and Utah, and collecting similar data as in the pilot. In total, 814 children with SB, aged 3-6 years, were identified, of whom 366 (44.9%) were eligible to participate instead of 368 (45.2%). Medical records were abstracted on 188 (51.4%) of eligible participants. There were unexpected methodological challenges that arose due to differences in the sampling plan between sites, primarily due to differences in IRB permissions for study selection and to some participants’ perception of the potential for adverse outcomes as a result of participation.

Differences in the approved sampling design between Utah and Arizona sites presented variation in study methodology. First, the sampling frame in Utah was a more comprehensive list of the eligible families than that of Arizona. Eligible participants in Utah were derived from a population-based state surveillance system, whereas those in Arizona were identified through varying sources that included a birth defects monitoring program, multispecialty clinics, and hospital databases. Therefore, Utah had greater target population representation in their sample and findings from their site may have greater generalizability and relevance to the interested Utah population. Second, Arizona was not allowed to actively recruit from their list of eligible participants. Although a common study methodology was agreed on between sites to evaluate parents and their children with SB, the difference in the processes permitted by the IRBs for the identification, ascertainment, and recruitment of eligible children with SB impacted Arizona’s participation rate. Utah’s population-based surveillance program within the Utah Department of Health was approved by the IRB to identify eligible children with SB and invite the parents directly to participate in this study. The IRB in Arizona permitted access to medical records of children whose parents were not contacted to participate; however, a challenge for the Arizona site was the inability to mail letters or directly recruit parents and their children from the SB specialty clinic. Thus, one lesson learned from this multisite study was that variance in sampling frame can impact study methodology and participant selection. In this research, the difference in permissible methods of contacting eligible participants affected the response rate in Arizona. The external validity of findings from Arizona is limited since the proportion of eligible participants who participated was low.

There are unique challenges to participating in research for individuals who have complex conditions. SB requires multidisciplinary care and services, which can be time-consuming and cumbersome for parents. Parents must devote significant time and resources to finding and utilizing health and educational services needed by their children. These demanding tasks may reduce the opportunities for participation in research, which could be seen as having no immediate or long-term benefit for their child. Yet having a child with a severe impairment—or the time constraint that might result—does not explain the differential recruitment in the current study because this factor is not likely to differ between sites or states. In addition, those who found the in-person assessment to be too time-consuming had the option of participating by phone survey, which required less time commitment, and this option was offered at both sites. Given that the study was designed to be population-based, the different outcomes in recruitment in the two states may be the result of both the contrasting interpretations by the local IRBs of rules created to protect human subjects and the differences in the racial/ethnic composition and geographic distribution of the population between the two states.

Utah’s population is relatively homogeneous, with non-Hispanic whites representing 79% and Hispanics 13% of the population [30]. Geographically, 75.5% of Utah’s population lives within 50 miles north and south of Salt Lake City (known as the Wasatch Front) where the tertiary and subspecialty pediatric clinical services are located [31]. With medical care for children and adolescents centrally located within the state and the majority of residents living along the Wasatch Front, parents may be more likely to participate in studies that are based where this care is provided.

In Arizona, 90% of the population lives in urban areas, primarily in and around Phoenix and Tucson. Arizona has a unique racial/ethnic distribution of 57% non-Hispanic white, 31% Hispanic, and 4% Native American. Most pediatric specialty services can be accessed only at facilities in the Phoenix and Tucson metro areas. Since the population in Arizona lives primarily in the urban areas where the specialty clinics are located, it is more likely that there may be a cultural explanation to the low participation rate in Arizona. The principal investigator in Arizona observed that some Hispanic parents, when approached to participate by study recruiters, were reluctant to test their children because they felt that their children did not need another medical or cognitive label (author SR, personal communication, September 20, 2012). The fear of a stigmatizing label that could arise from poor performance on cognitive testing was likely a deterrent for these families. Additionally, if the principal investigator, a person trusted and known by the patients, explained the benefits and risks of the study, they were more likely to agree to participate. Feelings of discomfort and fear of loss of privacy have been recognized in other studies as reasons for low participation [32]. Some of these issues may apply to the population in this study.

Recruiting participants to engage in research can be demanding and some of the challenges are highlighted here. Difficulties recruiting and retaining individuals with specific conditions or diseases to participate, for example, in clinical trials and behavioral interventions have been discussed elsewhere [32-36]. For a study that is dependent on the successful recruitment of representative samples, considering the challenges of recruitment at participating sites early in the planning stage may have a positive impact. The researchers may be aware of the specific factors and contexts that may challenge their recruitment efforts and address what can be done to counteract these. Considering the potential for variation in project interpretation by local IRBs early in the development phase may also contribute to a representative picture of the populations in the sites that participate.

### Conclusion

A total of 101 children aged 3-6 years and their families participated in this project in Arizona and Utah. Parents completed a survey that inquired about their child’s medical status, development and learning, nutrition and physical activity, mobility, general health, and family functioning. Medical records were abstracted for demographics, clinical characteristics, inpatient and outpatient encounters, and surgical history. Children were assessed in the areas of social and cognitive development and visual/motor skills. Additionally, families were assessed in the areas of family functioning, child behavior and personality, self-care, mobility and functioning, and health and well-being. It is expected that findings from these assessments will highlight areas of deficit that may impact the development of the child and their success in school. Knowledge of these deficits and development of plans to address them may support a more developmentally appropriate trajectory for the population of children affected by SB.

## Abbreviations

BBCS-R: Bracken Basic Concept Scale ‒ Receptive
CDC: Centers for Disease Control and Prevention
DAS-2: Differential Abilities Scale 2nd Edition
FAD: McMaster Family Assessment Device
IRB: institutional review board
NEPSY: NEuroPSYchological Assessment
PEDI: Pediatric Evaluation Disability Index
SB: spina bifida
WRAVMA: Wide Range Assessment of Visual Motor Abilities
